# Supplementary material for: Hybrid Composite Membrane of Phosphorylated Chitosan/Poly (Vinyl Alcohol)/Silica as a Proton Exchange Membrane
Source: Membranes (Basel). 2021 Aug 31;11(9):675. doi: 10.3390/membranes11090675 (PMC8470232; doi:10.3390/membranes11090675)
Supplement: Supplementary file 1 [file membranes-11-00675-s001.zip › membranes-1268211-supplementary.pdf]

membranes

MDPI

Supplementary Information

Hybrid Composite Membrane of Phosphorylated Chitosan/Poly (Vinyl Alcohol)/Silica as a Proton Exchange Membrane

Nur Adiera Hanna Rosli [1], Kee Shyuan Loh [1,*], Wai Yin Wong [1], Tian Khoon Lee [2] and Azizan Ahmad [2]

[1] Fuel Cell Institute, Universiti Kebangsaan Malaysia, 43600 UKM Bangi, Selangor, Malaysia; adierahanna@gmail.com (N.A.H.R.); waiyin.wong@ukm.edu.my (W.Y.W.)
[2] Faculty of Science and Technology, Universiti Kebangsaan Malaysia, 43600 UKM Bangi, Selangor, Malaysia; edison_tiankhoon@hotmail.com; azizan@ukm.edu.my
* Correspondence: ksloh@ukm.edu.my

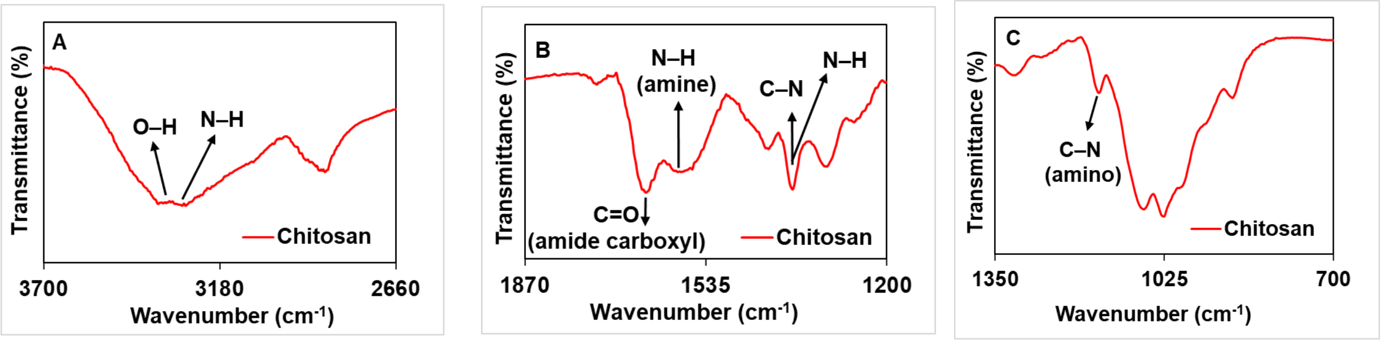

Figure S1. (**A**, **B** and **C**) The enlarged images of the FTIR spectra of chitosan.

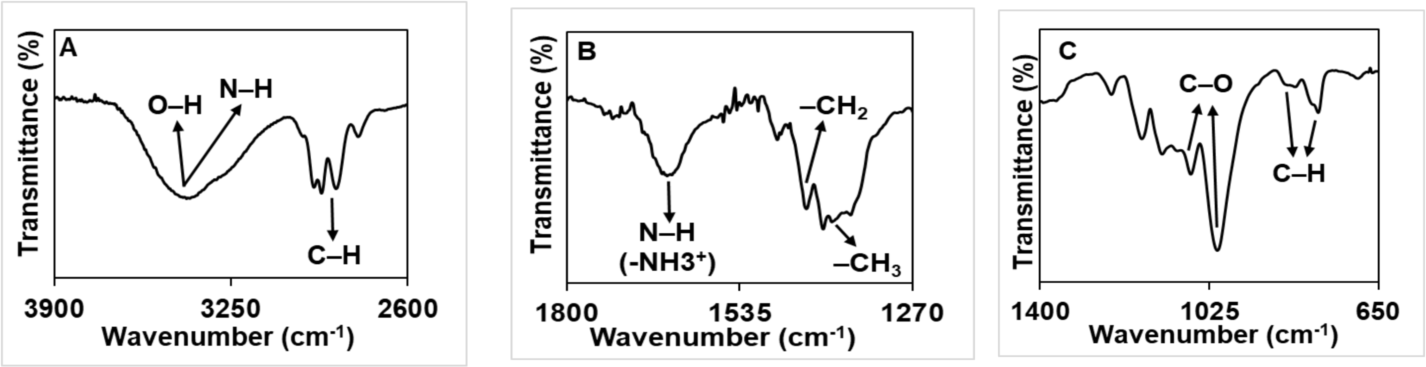

Figure S2. (**A**, **B** and **C**) The enlarged images of the FTIR spectra of NMPC/PVA composite membranes.

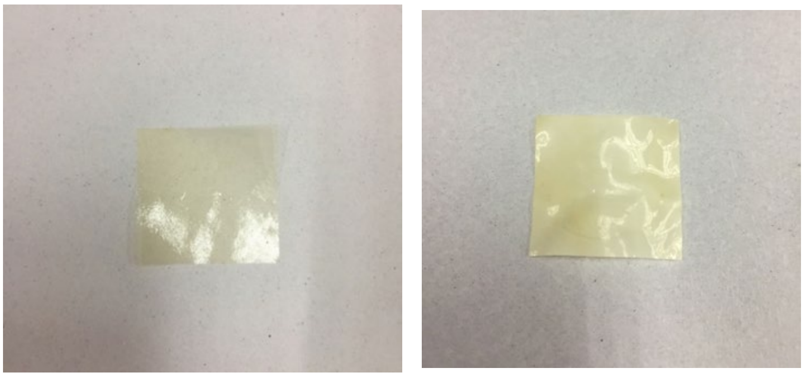

(**a**) (**b**)

Figure S3. Digital images of (**a**) NMPC membrane and (**b**) NMPC/PVA composite membrane.

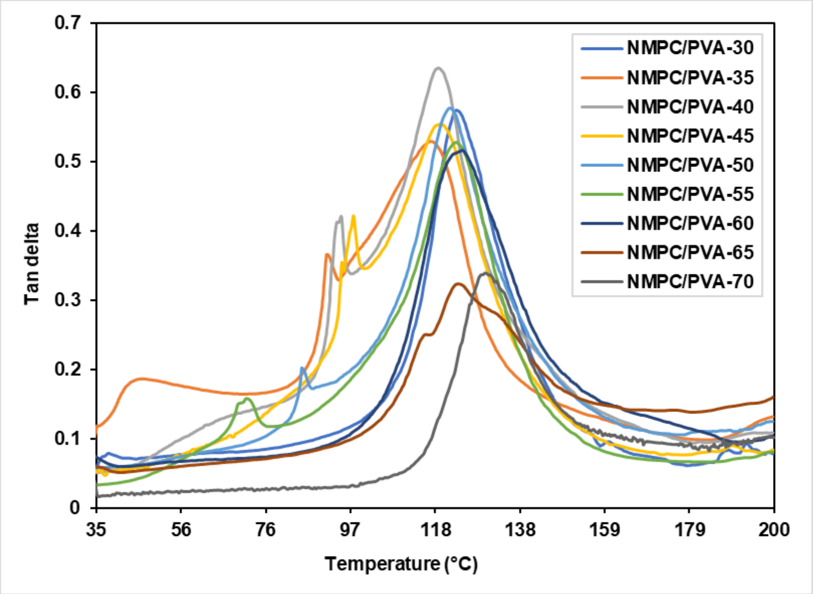

Figure S4. Comparisons of tan delta curves for NMPC/PVA membranes with different compositions.

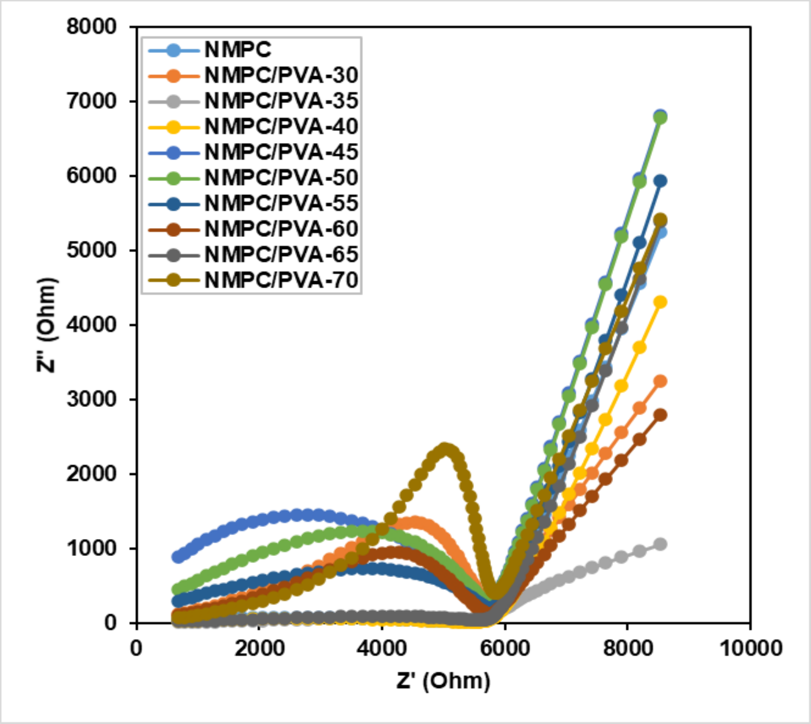

Figure S5. Nyquist plots for the NMPC membrane and NMPC/PVA composite membranes with different compositions.

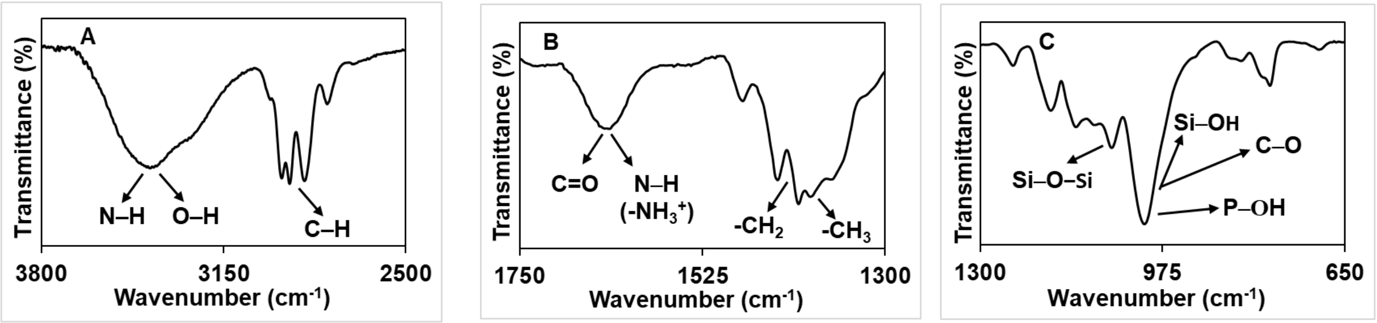

Figure S6. (**A**, **B** and **C**) The enlarged images of the FTIR spectra of NMPC/PVA-SiO_2 composite membranes.

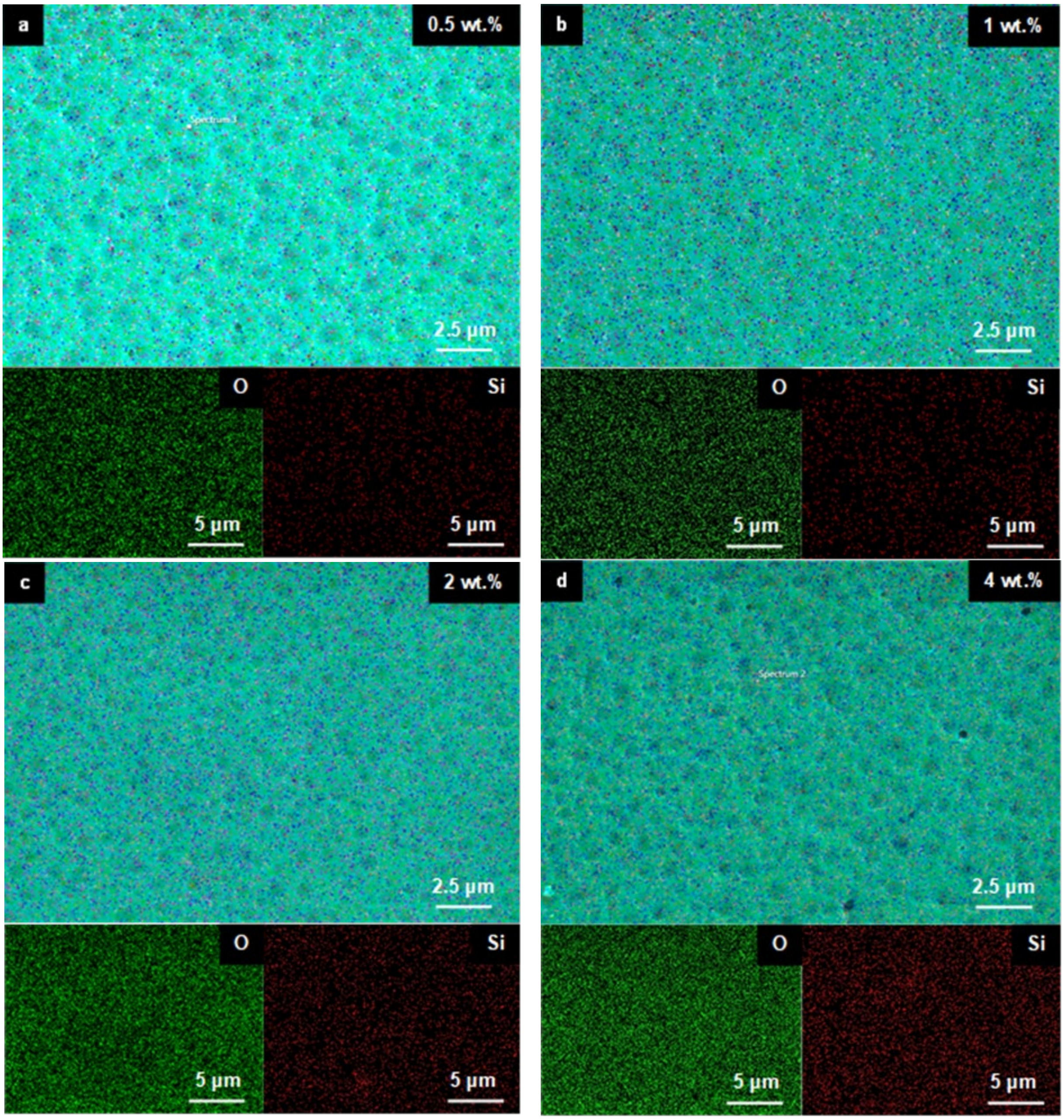

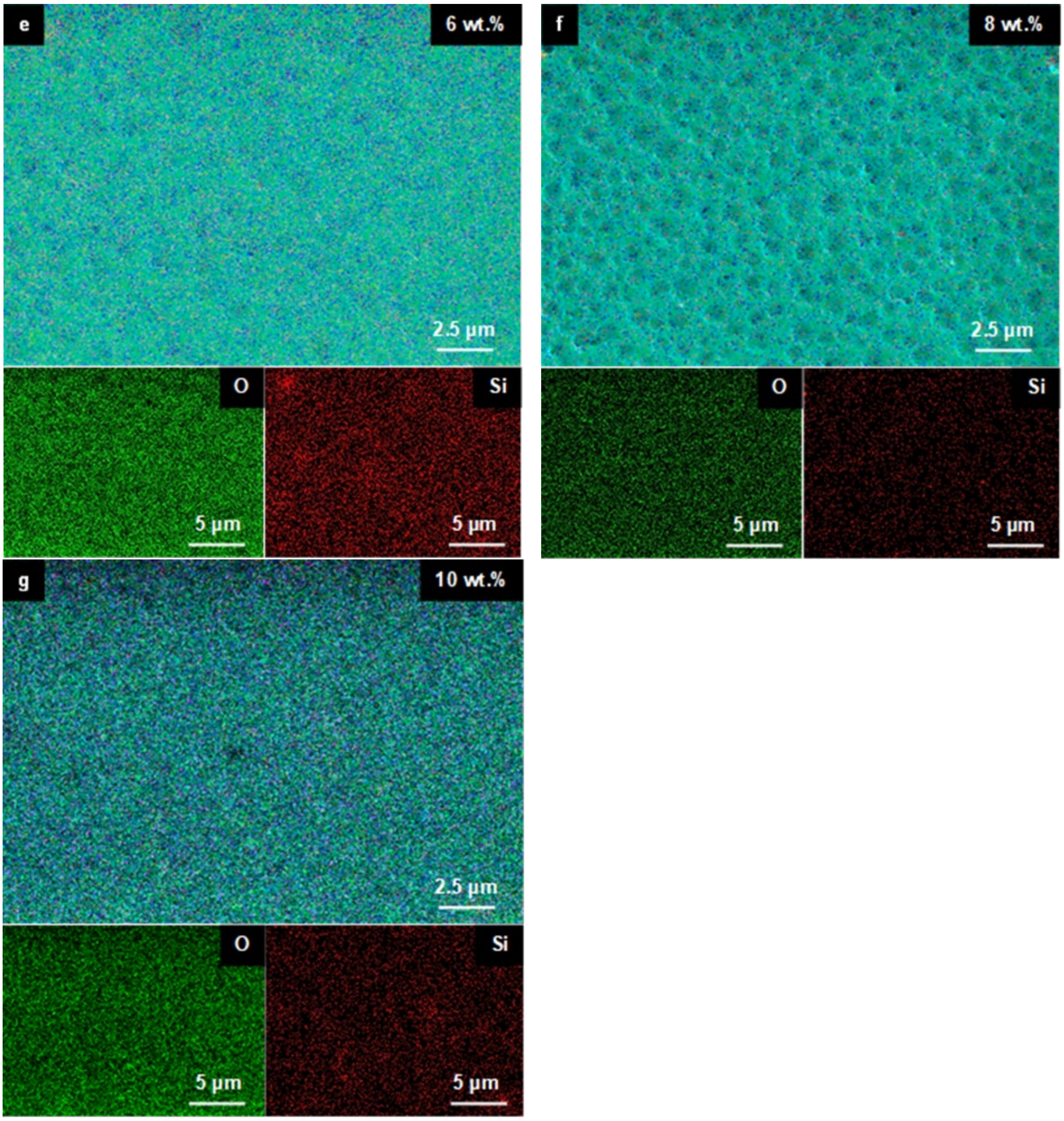

Figure S7. FESEM micrographs of the elemental mapping of the (**a**) NMPC/PVA-SiO_2 (0.5 wt.%), (**b**) NMPC/PVA-SiO_2 (1 wt.%), (**c**) NMPC/PVA-SiO_2 (2 wt.%), (**d**) NMPC/PVA-SiO_2 (4 wt.%), (**e**) NMPC/PVA-SiO_2 (6 wt.%), (**f**) NMPC/PVA-SiO_2 (8 wt.%) and (**g**) NMPC/PVA-SiO_2 (10 wt.%) composite membranes.

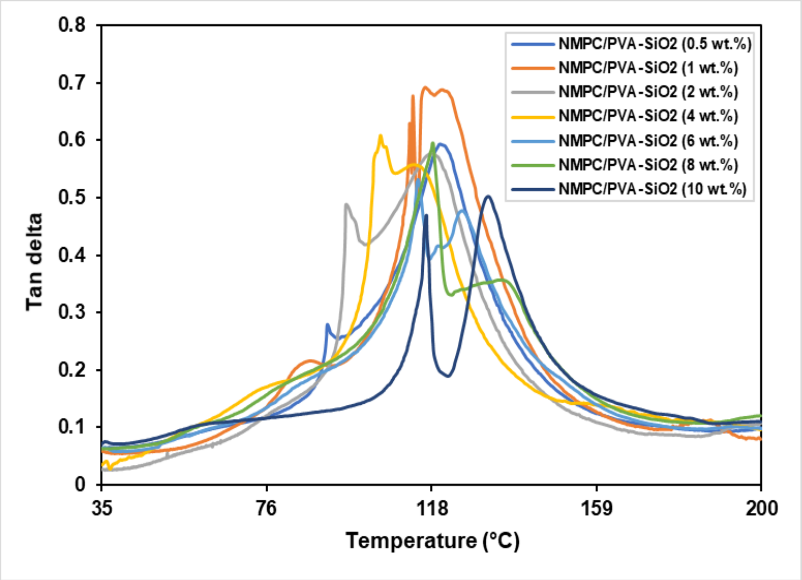

Figure S8. Comparisons of tan delta curves for NMPC/PVA-SiO_2 membranes with different compositions.

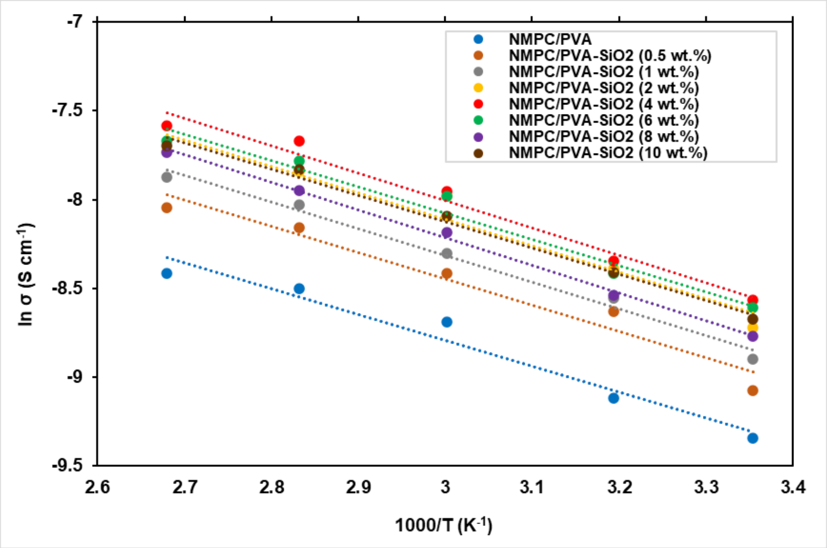

Figure S9. Arrhenius plots of the NMPC/PVA and NMPC/PVA-SiO_2 composite membranes.

Table S1. Thermal stability analysis of NMPC membrane and NMPC/PVA composite membranes with different compositions.

Membrane Sample	Degradation Temperature Range (°C)			Weight Loss (%)			Residual
	I	II	III	I	II	III	
NMPC	50.5–70.6	200.1–220.4	-	17	38	-	45
NMPC/PVA-30	80.3–101.2	230.5–302.4	371.6–460.9	10	23	54	13
NMPC/PVA-35	80.5–100.9	229.8–300.7	372.1–460.4	9	23	51	17
NMPC/PVA-40	81.2–100.4	230.6–301.5	371.9–461.5	9	27	47	17
NMPC/PVA-45	80.3–101.1	231.2–301.4	370.6–459.8	10	25	48	17
NMPC/PVA-50	80.6–100.8	231.4–300.9	371.3–461.5	10	20	53	17
NMPC/PVA-55	81.4–101.6	232.6–299.8	370.5–460.7	9	26	45	20
NMPC/PVA-60	80.8–100.7	231.3–301.2	369.7–459.6	9	30	39	22
NMPC/PVA-65	79.7–100.5	230.3–300.3	370.8–460.3	10	28	37	25
NMPC/PVA-70	79.9–101.3	229.9–300.6	371.2–460.1	9	27	42	22

Table S2. Thermal stability analysis of NMPC/PVA-50 composite membrane and NMPC/PVA-SiO_2 composite membranes with different compositions.

Membrane Sample	Degradation Temperature Range (°C)			Weight Loss (%)			Residual
	I	II	III	I	II	III	
NMPC/PVA-50	80.6–100.8	231.4–300.9	371.3–461.5	10	20	53	17
NMPC/PVA-SiO2 (0.5 wt.%)	102.4–148.9	240.7–301.4	381.5–470.9	10	24	46	20
NMPC/PVA-SiO2 (1 wt.%)	101.9–149.6	239.9–302.7	382.6–470.4	11	33	32	24
NMPC/PVA-SiO2 (2 wt.%)	100.5–150.2	240.8–302.5	381.3–469.8	11	30	35	24
NMPC/PVA-SiO2 (4 wt.%)	100.8–149.8	241.5–300.4	380.6–468.9	10	30	36	24
NMPC/PVA-SiO2 (6 wt.%)	101.7–150.7	241.6–301.9	381.1–472.2	10	28	37	25
NMPC/PVA-SiO2 (8 wt.%)	102.6–149.5	242.9–299.7	380.5–470.7	11	34	27	28
NMPC/PVA-SiO2 (10 wt.%)	101.3–150.9	243.3–302.2	379.7–469.6	9	28	33	30
